# SPHK1在调节小细胞肺癌多药耐药中的作用

**DOI:** 10.3779/j.issn.1009-3419.2014.11.01

**Published:** 2014-11-20

**Authors:** 兰 杨, 洪林 胡, 颖 邓, 义凤 白

**Affiliations:** 610000 成都，四川省人民医院肿瘤科 Department of Oncology, Sichuan Academy of Medical Sciences & Sichuan Provincial People's Hospital, Chengdu 610000, China

**Keywords:** SPHK1, 多药耐药, 肺肿瘤, Sphingosine kinase 1 (SPHK1), Multi-drug resistance, Lung neoplasms

## Abstract

**背景与目的:**

小细胞肺癌约占全部肺癌的15%，化疗是其主要的治疗方法之一，虽然早期对一线化疗方案敏感，但极易出现多药耐药而导致治疗失败。前期基因芯片发现SPHK1与小细胞肺癌的耐药性相关，本研究进一步探讨SPHK1在小细胞肺癌多药耐药中的作用。

**方法:**

首先通过QRT-PCR和Western blot从基因和蛋白水平检测化疗敏感细胞株H69及多药耐药细胞株H69AR中SPHK1的差异表达；转染siRNA下调H69AR细胞中的SPHK1的表达，通过CCK8检测细胞对各种化疗药物（ADM, DDP, VP-16）的敏感性变化，流式细胞仪检测细胞周期及凋亡的变化。同时收集小细胞肺癌化疗前组织和血液标本，将其分为化疗敏感组和耐药组，QRT-PCR检测小细胞肺癌患者血液标本中SPHK1的表达，免疫组化法检测小细胞肺癌患者组织标本中SPHK1的表达，分析SPHK1与小细胞肺癌患者预后相关性。

**结果:**

SPHK1在耐药细胞H69AR中的表达明显高于H69，下调H69AR中SPHK1的表达能够增加细胞对化疗药物的敏感性，促进细胞的凋亡，细胞周期发生G_0_/G_1_期阻滞，SPHK1在小细胞肺癌耐药患者中的表达较敏感患者明显增加，SPHK1的表达与患者的性别、年龄无关，与疾病的分期、对化疗的敏感性及生存时间密切相关，差异具有统计学意义（*P*＜0.05）。

**结论:**

SPHK1参与调节小细胞肺癌多药耐药，SPHK1可作为评估小细胞肺癌化疗敏感性及临床预后的潜在靶基因。

## Abstract (en)

**Background and objective:**

Lung cancer is the leading cause of cancer-related deaths worldwide. Approximately 15% of all histological types consist of small cell lung cancer (SCLC). Chemotherapy is one of the major treatment method. Though the current first-line standard chemotherapy regimen for SCLC is active in most SCLC cases, however the disease recurs shortly after the first successful treatment with multi-drug resistance (MDR) phenotype. Our previously study showed that SPHK1 was associated with MDR in SCLC. The aim of this study is to investigate the role of sphingosine kinase 1 (SPHK1) showed in small cell lung multi-drug resistance.

**Methods:**

Firstly, the analysis of QRT-PCR and Western blot were used to study differential expression of SPHK1 from mRNA and protein levels in both the H69 and H69AR cell lines. Then, Downregulation of SPHK1 by transfection with siRNA in H69AR. Moreover, the sensitivities of cells to chemotherapy drugs such as ADM, DDP, VP-16 were detected by CCK8 assay. The change of cell cycle and apoptosis rate were detected by flow cytometry. Meanwhile, expression of SPHK1 in clinical specimens were detected by QT-PCR and immunohistochemistry. Relation of SPHK1 expression with clinicopathological features and prognosis of patients was studied.

**Results:**

The expression of SPHK1 was significantly decreased in H69AR cells that in the H69 cells. The sensitivities of H69AR cells to chemotherapy drugs were increased when up-regulated the expression of SPHK1, enforced SPHK1 expression increased cell apoptosis and the cell cycle arrest in G_0_/G_1_ phase in H69AR cells, the expression of SPHK1 was not associated with gender, age, but significantly correlated with clinical stage, chemosensitivity and overall survival (*P* < 0.05).

**Conclusion:**

Our results suggest that SPHK1 is involved in the regulation of small cell lung cancer multi-drug resistance, SPHK1 may be as potentialtarget gene to evaluate the chemosensitivity and clinical prognostic for SCLC.

小细胞肺癌（small cell lung cancer, SCLC）的细胞倍增时间短，病情进展快，早期即发生血道和淋巴道转移，恶性程度在所有肺癌类型中最高。SCLC的治疗以放化疗为主，尽管80%患者早期对放、化疗呈现出较好的初始反应性，但很快即产生复发或病情进展。局限期患者5年生存率低于30%，广泛期患者5年生存率仅1%-2%^[1]^。SCLC的耐药，尤其是多药耐药（multi-drug resistance, MDR）的产生是其治疗失败的最重要原因^[2]^。因此，化疗抗药已成为目前SCLC临床治疗急需解决的问题之一。

鞘氨醇激酶-1（sphingosine kinase-1, SPHK1）是一种鞘氨醇代谢酶，于1998年首先在大鼠肾细胞中被提取出来，相对分子质量为49, 000。目前己发现，在人类和小鼠组织中存在Sphkl和Sphk2两种异构体。人类细胞中，Sphkl基因位于17号染色体，Sphk2位于19号染色体。Sphkl和Sphk2有着不同的生物学功能及调控机制。SPHK1分子主要分布在脑、心脏、肺脏、肝脏、脾脏和造血免疫系统，与多种肿瘤细胞的过度增殖有关，参与调节细胞的多种生物学功能。SPHK1的过度表达能够提高细胞周期从G_1_期向S期转化的速率，减少细胞倍增的时间，从而对细胞周期调控发挥作用^[3]^。研究人员^[4]^在前列腺癌方面的研究中发现，上调SPHK1的表达能降低羟喜树碱诱导的前列腺癌PC3细胞凋亡。Sobue等^[5]^的研究发现SPHK1由于其在白血病患者中呈高表达，因此SPHK1既是预测白血病对柔红霉素敏感性的一个好的标志物，又是一个白血病的潜在治疗靶点，在柔红霉素诱导的细胞毒性反应中发挥着重要的作用。研究表明，SPHK1可能与某些肿瘤的发生发展密切相关，但SPHK1对肿瘤耐药方面的研究还很少见，尤其在SCLC耐药中的作用目前国内外还未见相关的报道。我们前期通过基因表达谱芯片对SCLC耐药细胞H69AR和非耐药细胞H69中21, 522个基因进行分析，结果发现H69AR细胞中1, 131个基因表达下调^[6]^，包含SPHK1在内的1, 252个基因表达上调，本实验旨在进一步验证SPHK1在SCLC敏感和耐药患者及细胞株中的差异表达，以及其表达对SCLC化疗药物敏感性、细胞周期、凋亡及临床预后的影响。

## 1 材料与方法

### 1.1 标本来源

76例SCLC患者化疗前外周血液标本均来源于2007年1月-2013年10月四川省人民医院肿瘤科及呼吸内科，将其分为化疗敏感组（化疗5个-6个周期后肿瘤完全缓解或部分缓解）36例，化疗耐药组（化疗5个-6个周期后疾病稳定或进展者）40例，采用Dextran沉降法、标准的梯度密度离心的方法分离SCLC血液标本中有核细胞^[7]^，具体步骤如下：使用无菌采血针抽取SCLC外周静脉血至真空采血管（含EDTA抗凝）中，加入二分之一体积的6% DextranT-500/0.9% NaCl缓慢颠倒混匀，37 ℃水浴沉降30 min-45 min。待沉降完全后，小心将上层富血清吸入离心管中，2, 500 rpm/min离心30 min；去上清，收集管底沉淀，加入Trizol 1 mL移入去RNA酶的EP管中，放-80 ℃保存。76例患者均接受EP方案[足叶乙甙（VP 16）加顺铂（DDP）]化疗，本研究经本院伦理委员会批准，所有患者均签署知情同意书。随访：全部76例患者出院后均随访，随访方式为电话随访和复查随访，随访内容包括一般情况、临床症状及影像学检查。随访起点为确诊日期，末次随访时间为2014年1月31日，中位随访时间约24个月，至随访截止日，依然生存34例，死亡42例，无失访病例。

### 1.2 主要试剂

RPMI-1640培养基（Gibco）；胎牛血清（Gibco）；opti-MEM（Gibco）；Lipofectamine 2000；胰酶（0.02%EDTA）；顺铂、阿霉素和依托泊苷购自辉瑞公司；CCK8及凋亡检测试剂盒购自上海碧云天公司；Trizol RNA试剂盒、RT试剂盒、Taq酶、PCR试剂盒均购自Invitrogen公司，第1链cDNA合成试剂盒购自大连宝生生物公司；鼠抗人单克隆抗体SPHK1购自美国Santa Cruz公司；兔抗鼠IgG二抗购自基因（上海）生物有限公司。

### 1.3 主要仪器

超净工作台；细胞培养箱；高速冷冻离心机；蛋白电泳仪；酶标仪；荧光定量PCR仪（Eco）；FAC-Sort型流式细胞仪（Becton Dicksongon公司）。

### 1.4 细胞系

人SCLC敏感细胞株（H69）及对阿霉素耐药的细胞株（H69AR）均购自美国ATCC公司。

### 1.5 方法

#### 1.5.1 细胞培养和传代

应用RPMI-1640培养基做细胞扩增培养基，H69细胞株培养基含15%胎牛血清，H69AR细胞株培养基含20%胎牛血清。培养条件:在37 ℃、5%CO_2_细胞培养箱。

#### 1.5.2 实时荧光定量PCR分析SPHK1的mRNA表达

用Trizol RNA试剂盒提取H69、H69AR细胞株中总RNA，操作按照说明书进行；测量总RNA浓度，取1 μg RNA按逆转录合成试剂盒说明合成cDNA，然后以该cDNA为模板，PCR仪中扩增提取SCLC细胞中的RNA进行逆转录，采用SYBR Green染料法行实时荧光定量PCR。SPHK1及内参照GAPDH的引物序列见表 1。逆转录反应及PCR扩增参照试剂盒说明进行，以*SPHK1*基因的上下游引物进行PCR扩增，PCR反应在实时定量PCR反应仪上进行；反应条件：95 ℃ 30 s，（95 ℃ 5 s, 60 ℃ 30 s）^*^40 cycles，4 ℃。反应结束后确认Real Time PCR的扩增曲线和融解曲线，进行PCR定量时制作标准曲线。基因的表达量F=2^-∆∆Ct^，三次独立实验后得到的数据运用公式RQ=2^-∆∆Ct^的方法进行分析。△△ct=（待测样品的目的基因的Ct的平均值-待测样本的看家基因的Ct的平均值）－（对照样品的目的基因的Ct的平均值-对照样本的看家基因的Ct的平均值）。

**1:** SPHK1及内参照GAPDH的引物序例 Primer sequence of SPHK1 and GAPDH

Gene	Primer
*SPHK1*	5’-ATGATCCTCTTGCAACACG-3’
	5’-CTGCAAGAGGCAAGGAAG-3’
*GAPDH*	5’-GGAAGGACTCATGACCACAGTCC-3’
	5’-TCGCTGTGAAGTCAGAGGAGACC-3’

#### 1.5.3 脂质体介导siRNA转染H69AR细胞

将合成的靶向作用于SPHK1的SiRNA（RNAi#1:GGCTGAAATCCCTTCACG; RNAi#2: GGGCAAGGCCTTGCAGCTC）寡核苷酸序列及阴性对照序列（NC），转染入H69AR细胞。转染前一天，将适当细胞浓度的H69AR细胞悬液接种于六孔培养板中，培养24 h，使细胞生长密度达60%左右。将siRNA- lipofectamine 2000混合液（500 μL/孔）加入含有细胞及1.5 mL/孔Opti-MEM培养液的培养板中，轻轻摇晃，使之混合。细胞板置于37 ℃、5%CO_2_培养箱中培养4 h-6 h后换新鲜培养基。转染后24 h检测基因表达的变化，48 h后检测蛋白的表达。

#### 1.5.4 Western blot分析SPHK1蛋白表达

提取细胞总蛋白（凯基全蛋白提取试剂盒），BCA法蛋白定量。制备电泳凝胶，每孔中加样50 μg蛋白，经10%SDS-PAGE后，电转移至PVDF膜。用5 g脱脂奶粉/100 mL TBST的封闭液中室温封闭2 h。TBST漂洗3次，每次5 min，加入鼠抗人SPHK1单克隆（1:200）孵育，4 ℃过夜。TBST漂洗3次，每次5 min，用HRP标记的兔抗鼠IgG（1:5, 000）孵育，37 ℃，45 min。TBST漂洗3次，每次5 min。加入显色液，暗室曝光10 s-10 min，显影。

#### 1.5.5 CCK8法检测药物敏感性

将对数生长期的细胞按5×10^3^个/孔接种于96孔培养板中，设空白对照孔。培养24 h后，将化疗药物加入培养基中并进行倍比稀释，按不同浓度分别加入指定的孔中，并设不加药物的阳性对照孔，每个药物浓度、空白对照孔、阳性对照孔均设5个复孔。细胞在含药培养基中培养24 h后，加入CCK8反应液，在37 ℃、5%CO_2_培养箱中培养1 h-4 h后在酶标仪上测450 nm吸光度。测得的（每个药物浓度的OD值的平均值-空白孔平均值）/不含药物的阳性对照组平均值即为每个药物浓度下细胞的存活率。重复实验3次，根据各个药物浓度细胞存活率，作对数曲线。

#### 1.5.6 细胞凋亡检测

对数生长期的细胞以4×10^5^/孔接种于6孔板中；37 ℃培养48 h；收集细胞，PBS洗涤2次；细胞重悬于100 μL含Annexin V-FITC和0.5 μg PI的结合缓冲液（10 mM HEPES pH 7.4, 0.15 M NaCl, 5 mM KCl, 1 mM MgCl_2_, 1.8 mM CaCl_2_）中；光室温孵育15 min；加入400 μL结合缓冲液；流式细胞仪分析。

#### 1.5.7 细胞周期检测

取对数生长期的细胞，用0.25%胰蛋白酶和0.02%EDTA消化细胞，PBS洗2次，用75%乙醇冰浴固定24 h，然后用含1%BSA的PBS充分混匀洗涤2次，PI染色后进行流式细胞仪测定并用Cell Quest软件分析各组细胞群体在细胞周期各个时相的分布比例。

### 1.6 统计学方法

所有实验所得数据采用SPSS 13.0统计软件进行分析，采用*t*检验或*One*-*way* ANOVA方法进行统计分析，以Mean±SD表示。SPHK1的表达与各临床病理参数之间的关系使用*Chi*-*Square*检验，生存分析用*Kaplan*-*Meier*法，所有统计结果均以*P*＜0.05为差异具有统计学意义。

## 2 结果

### 2.1 SPHK1在临床组织标本中的表达

免疫组织化学方法检测SPHK1在76例SCLC组织标本及癌旁组织中的表达，其中男性35例，女性41例；患者年龄30岁-75岁，中位年龄50岁，50岁以下患者41例，50岁及以上患者35例；局限期患者34例，广泛期42例。对化疗敏感者28例，对化疗耐药者48例，结果提示与癌旁组织比较（图 1A），SPHK1在SCLC组织中阳性表达位于细胞胞浆和胞膜（图 1B），在SCLC组织中阳性表达率为69.74%（53/76），在癌旁组织中的阳性表达率为8.70%（4/46），差异具有统计学意义（*P*＜0.01）。

**1:**
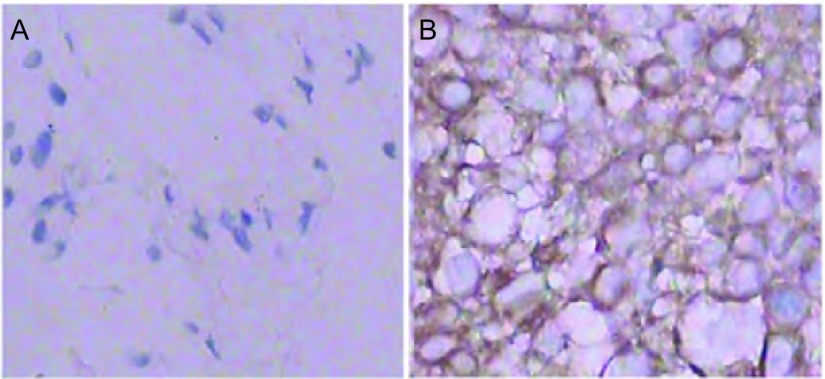
免疫组织化学法检测SPHK1在SCLC癌旁组织（A）及癌组织中（B）的表达（×400） The expression of SPHK1 was presented in the para-carcinoma tissues (A)and carcinoma tissues (B) (×400)

### 2.2 SPHK1在SCLC组织中的表达及其与临床病理特征的关系

SPHK1在男性患者中的阳性表达率为74.29%（26/35），在女性患者中的阳性表达率为65.85%（27/41），两者的SPHK1阳性表达率差异无统计学意义（χ^2^=0.636, *P*=0.425）。SPHK1在不超过50岁的患者中阳性表达率为70.73%（29/41），在50岁以上患者中阳性表达率为68.57%（24/35），两者的SPHK1阳性表达率无统计学意义（χ^2^=0.042, *P*=0.838）。SPHK1在局限期（LD）患者中的阳性表达率为52.94%（18/34），在广泛期（ED）患者中的阳性表达率为83.33%（35/42），两者的SPHK1阳性表达率差异具有统计学意义（χ^2^=8.224, *P*=0.004）。SPHK1在化疗敏感患者中的阳性表达率为46.43%（13/28），在化疗耐药患者中的阳性表达率为83.33%（40/48），两者的SPHK1阳性表达率差异具有统计学意义（χ^2^=11.412, *P*=0.001)。SPHK1在存活患者中的阳性表达率为47.06%（16/34），在死亡患者中的阳性表达率为88.10%（37/42），两者的SPHK1阳性表达率差异具有统计学意义（χ^2^=14.993, *P*＜0.001）（表 2）。

**2:** SPHK1的阳性表达与患者临床特征的关系 Association of SPHK1 with clinical parameters

Patients characteristics	SPHK1 expression		*χ*^2^	P^*^
	Low	High^#^		
All cases (*n*=76)	23	53		
Age			0.042	0.838
＜50	12	29		
≥50	11	24		
Gender			0.636	0.425
Male	9	26		
Female	14	27		
Disease stage			8.224	0.004
Limited disease (LD)	16	18		
Extensive-stage disease (ED)	7	35		
Response to chemotherapy			11.412	0.001
Response	15	13		
Refractory	8	40		
Survival state (3 mo-38 mo)			14.993	＜0.001
Survival	18	16		
Death	5	37		
^#^The median expression level was used as the cutoff. Low expression of SPHK1 in 23 patients was classified as values of 2^-△△ct^ below 1.0.High SPHK1 expression in 53 patients was classified as values of 2^-△△ct^ above 1.0. ^*^For analysis of correlation between of SPHK1 levels and clinical features, *Chi*-*square* Test were used. Results were considered statistically significant at *P*＜0.05.				

### 2.3 SPHK1在SCLC细胞株及血液标本中的表达

如图 2A所示，QRT-PCR结果显示H69AR细胞株中SPHK1的mRNA表达较H69细胞明显升高，差异具有统计学意义（*P*=0.001）。Western blot结果也显示在耐药株H69AR中的SPHK1蛋白的表达较敏感株H69升高（*P*=0.007）（图 2B）；QRT-PCR结果提示化疗耐药患者血液标本中SPHK1的表达明显高于化疗敏感的患者，差异具有统计学意义（*P*＜0.001）（图 2C）。

**2:**
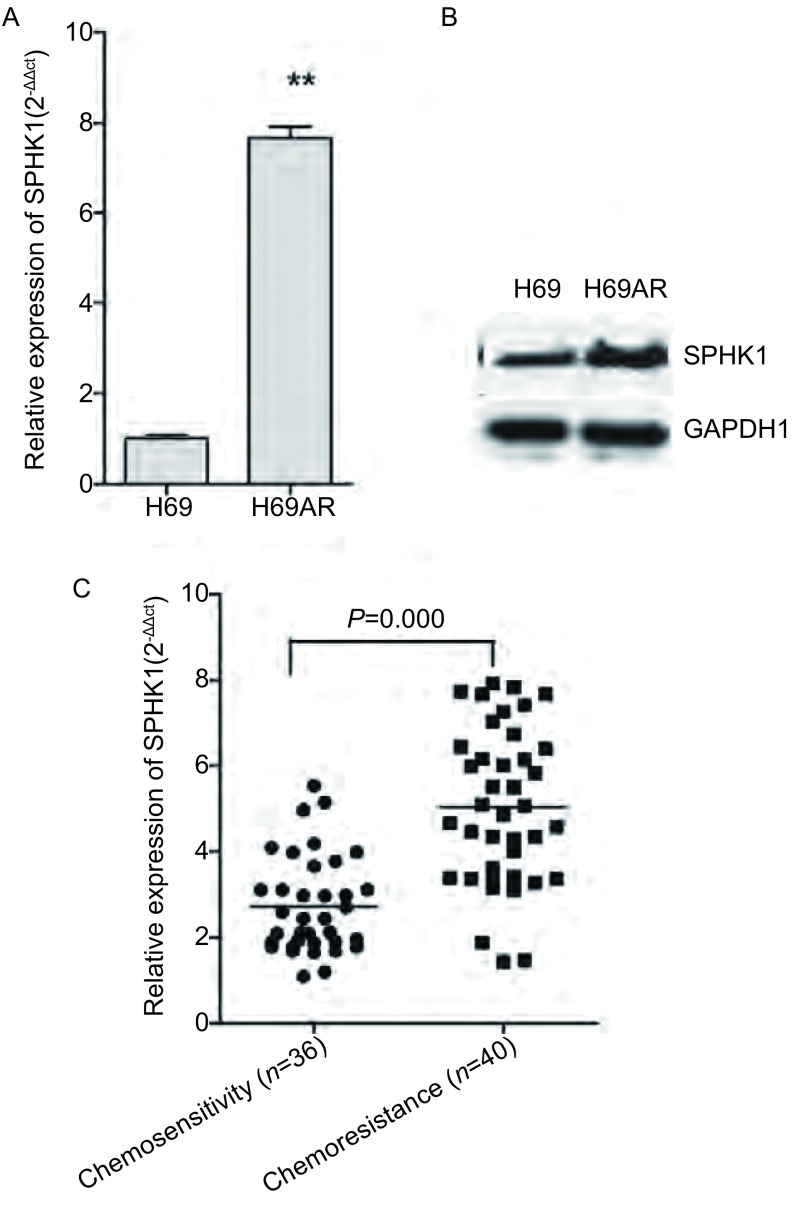
QRT-PCR和Western blot在mRNA水平（A）和蛋白水平（B）检测H69及H69AR细胞中SPHK1的表达；C：化疗耐药患者与化疗敏感的患者血液标本中SPHK1的差异表达，^**^与H69组比较，*P*＜0.01。 The expression of SPHK1 mRNA (A) and protein (B) were detected in H69 and H69AR by QRT-PCR and Western blot; C: The differential expression of SPHK1 in chemosensitivity and drug resistance patients. ^**^compare with H69, *P* < 0.01.

### 2.4 下调SPHK1的表达后细胞对化疗药物敏感性的变化

通过SiRNA下调H69AR细胞株中SPHK1的表达，通过QRT-PCR检测Si-SPHK1的干扰效率，如图 3A所示，H69AR细胞转染SPHK1的小干扰RNA后，与空白对照H69AR及阴性对照H69AR-NC比较，H69AR-Si-SPHK1细胞中SPHK1的表达下降76.3%，差异具有统计学意义（*P*＜0.001）。CCK8检测化疗药物敏感性的变化，结果提示下调SPHK1的表达后，细胞对化疗药物DDP，ADM及VP-16的敏感性明显增加，细胞的存活率降低，差异具有统计学意义（*P*＜0.01）（图 3B-图 3D）。

**3:**
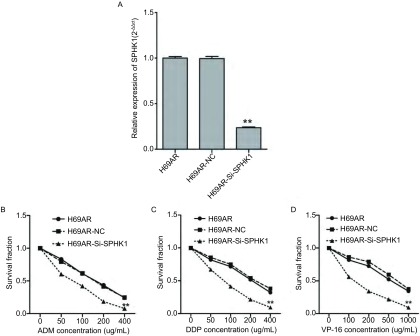
下调SPHK1的表达后细胞对化疗药物敏感性的变化。A：细胞转染SPHK1的SiRNA后，在mRNA水平检测其干扰效率。H69AR为空白对照；H69AR-NC为阴性对照；H69AR-Si-SPHK1为转染SPHK1下调H69AR细胞中SPHK1的表达；CCK8检测下调H69AR中SPHK1的表达后, 细胞对化疗药物ADM（B），DDP（C）及VP-16（D）的敏感性的变化。^**^与H69AR及H69AR-NC组比较，*P*＜0.01. DDP：顺铂；ADM：阿霉素；VP-16：依托泊苷。 The sensitivities of cells to chemotherapy drugs were measured after H69AR cells transfected with Si-SPHK1. A: Expression of SPHK1 after transfection of SPHK1 siRNA in H69AR cells. The sensitivities of cells to chemotherapy drugs ADM (B), DDP (C) and VP-16 (D) were measured after H69AR cells transfected with Si-SPHK1 or mock by CCK-8 assay. ^**^compare with H69AR and H69AR-NC, *P* < 0.01. DDP: Cis-platinum; ADM: Adriamycin; VP-16: Etoposide.

### 2.5 下调SPHK1的表达后细胞周期和凋亡的变化

流式细胞技术检测细胞周期的变化，下调H69AR细胞中SPHK1的表达后，H69AR-Si-SPHK1组细胞周期G_0_/G_1_期细胞较H69AR组及H69AR-Si-NC组明显增多，S期细胞明显减少。结果提示下调SPHK1使细胞周期发生G_0_/G_1_期阻滞（图 4A，*P*＜0.001）。通过SiRNA技术下调H69AR细胞中SPHK1的表达后H69AR-Si-SPHK1组细胞的凋亡率为（24.32%±0.406%）明显高于空白对照H69AR（5.14%±0.067, 2%）及阴性对照H69AR-NC（6.07%±0.087, 6%）组细胞，差异具有统计学意义（图 4B，*P*＜0.01），提示下调SPHK1的表达能明显促进了H69AR细胞的凋亡。

**4:**
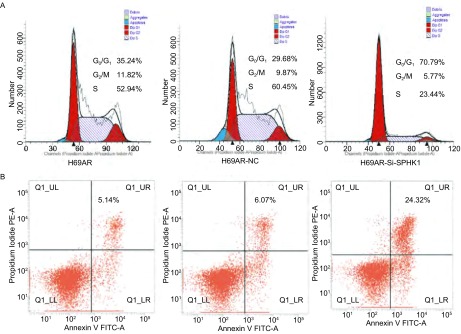
下调SPHK1的表达后流式细胞术检测细胞周期（A）和凋亡（B）的变化 The cell cycle (A) and apoptosis rate (B) was detected by flow cytometric analysis after transfected with Si-SPHK1

### 2.6 SCLC患者生存及预后分析

采用*Kaplan*-*Meier*法估计患者生存时间，结果发现患者的生存时间与性别和年龄无相关性，差异无统计学意义（*P*＞0.05）；疾病的分期，患者对化疗的敏感性及SPHK1的表达与患者的生存时间相关。局限期患者的生存时间长于广泛期患者，差异具有统计学意义（χ^2^=7.796，*P*=0.005，图 5A）；对化疗敏感者的生存时间长于耐药患者，差异具有统计学意义（χ^2^=8.985，*P*=0.003，图 5B）；SPHK1低表达患者的生存时间长于高表达患者，差异具有统计学意义（χ^2^=27.385，*P*＜0.001，图 5C）。

**5:**
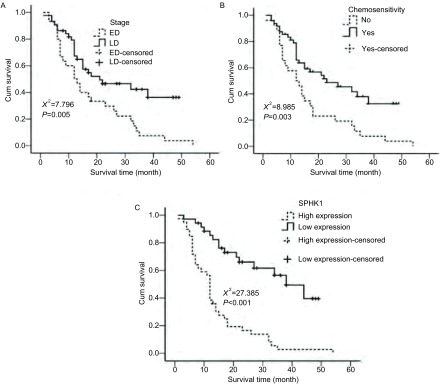
*Kaplan*-*Meier*法估计疾病分期（A）、化疗敏感性（B）及SPHK1的表达（C）与患者生存时间的关系。LD：局限期；ED：广泛期。 The relationship of survival time with disease stage (A), chemosensitivity (B) and expression of SPHK1 (C) by *Kaplan*-*Meier* assay. LD: Limited disease; ED: Extensive-stage disease.

*Cox*回归分析患者的性别、年龄、疾病分期、化疗敏感性及SPHK1的表达与患者预后的关系，发现疾病分期和SPHK1可作为独立的预后因子，分期越晚的相对危险度2.261，95%相对危险度的可信区间为（1.225-4.172），差异具有统计学意义（*W*=6.813, *P*=0.009）；SPHK1高表达的相对危险度为5.431，95%相对危险度的可信区间为（2.484-11.875），差异具有统计学意义（*W*=17.976, *P*＜0.001）（表 3）。

**3:** *Cox*回归分析疾病的分期、SPHK1的表达水平与NSCLC患者预后的关系 *Cox* regression analysis on relationship between disease stage, expression levels of SPHK1 and prognostic of NSCLC patients

Variable	B	SE	Wald	*P*	RR (95%CI)
Disease stage	0.816	0.313	6.813	0.009	2.261 (1.225-4.172)
Age	-0.181	0.419	0.185	0.667	0.835 (0.367-1.899)
Gender	-0.218	0.426	0.262	0.609	0.804 (0.349-1.854)
Chemosensitivity	0.282	0.425	0.440	0.507	1.326 (0.576-3.051)
Expression of SPHK1	1.692	0.399	17.976	＜0.001	5.431 (2.484-11.875)
NSCLC: non-small cell lung cancer.					

## 3 讨论

肺癌是当今世界对人类健康危害最大的恶性肿瘤之一，我国肺癌发病率增长迅速，多数学者把更多的研究目光集中在非小细胞肺癌（non-small cell lung cancer, NSCLC）治疗上，而SCLC的发病率却悄然增长，由于研究的关注度和力度明显不及前者，多年来治疗无明显突破^[8, 9]^。SCLC的治疗以全身化疗为主，联合放疗和手术为主要治疗手段。目前临床上化疗一般采取顺铂为基础的两药联合治疗方案，尽管SCLC患者对化疗敏感，但很快出现的MDR而导致化疗失败^[10]^。因此，如何克服SCLC的耐药性，成为临床上治疗SCLC及提高患者生活质量、延长生存时间急需解决的重要问题。SCLC的耐药机制非常复杂，常有多种因素参与其中，现己认识到的耐药机制包括：①具有外排泵作用的膜蛋如ABC家族成员P-糖蛋白（P-glycoprotein, P-gp）、肺耐药蛋白（lungresistance-related protein, LRP）等过度表达；②细胞内酶系统异常，如拓扑异构酶、谷胺酞转肤酶等；③细胞抗凋亡作用增强，如抗凋亡基因Bcl-2、cmyc等过度表达；④细胞修复系统增强。这些途径中的关键基因在遗传学水平及表观遗传水平的改变均可诱发肿瘤细胞形成耐药表型^[11]^。

SPHK1为S1P产生的限速酶，S1P在脂质代谢过程中发挥重要作用，可间接的调控S1P的功能，如抑制细胞凋亡、调节粘附相关分子的表达、刺激细胞的增殖效应等^[12]^。在生物体内一些受体酪氨酸激酶以及多种生物活性剂都可激活SPHK1的表达，而被激活的SPHK1在生理条件下可以刺激细胞的增生，但SPHK1活性的异常激活将可能导致细胞的恶性增生及肿瘤的发生^[13]^。Tan等^[14]^的研究发现，SPHK1作为结肠癌独立预后因子，促进结肠癌的恶性进展。Meng等^[15]^通过QRT-PCR和免疫组化法检测膀胱癌组织和癌旁组织中SPHK1的差异表达，结果提示SPHK1在癌组织中的表达明显高于癌旁组织，SPHK1的表达于膀胱癌的组织学分级和临床分期相关，高表达SPHK1的5年生存率明显降低，SPHK1作为独立的预测膀胱癌预后差的指标。Malavaud^[16]^认为SPHK1过度激活所呈现的恶性表现可能是由于SPHK1的磷酸化以及由其引起的SPHK1的蛋白异位引起。SPHK1不仅在人多种恶性肿瘤中过表达，同时其参与到了肿瘤血管的形成，促进肿瘤细胞的增殖，抑制肿瘤细胞的凋亡以及在肿瘤细胞药物耐药中发挥重要的作用。血管的再生在实体瘤发展中占有重要作用，研究发现S1P能够直接作用于内皮细胞诱导血管形成，且其诱导能力远远比VEGF有效，同时还能增加人脐血管内皮细胞内的VEGF mRNA的表达，与VEGF发挥协同作用，共同促进肿瘤血管的生成^[17]^。新近的研究^[18]^发现，SPHK1的过表达不仅能够抑制肿瘤细胞的凋亡，同时SPHK1的高表达还能增加细胞由G_0_/G_1_期阻滞。Rosa等^[19]^通过检测直肠癌患者体内的SPHK1的表达，结果发现在先天性或获得性对西妥昔单抗耐药的患者中SPHK1的表达是增高或者活化的。我们在前期对SCLC耐药细胞株和敏感细胞株高通量芯片筛选中发现耐药细胞株中SPHK1的表达明显增高，为了进一步验证芯片结果我们进一步从基因和蛋白水平检测了SCLC中SPHK1的表达，同时检测了对化疗敏感和耐药患者血液标本中SPHK1的差异表达，结果与芯片一致。随后通过SiRNA下调SPHK1的表达后，发现细胞对化疗药物的敏感性明显增加，下调SPHK1的表达后细胞凋亡明显增加，细胞周期发生G_0_/G_1_期阻滞，SPHK1的表达与患者的性别、年龄无相关性，差异无统计学意义；与患者对化疗药物的敏感性、疾病分期及生存期密切相关，差异具有统计学意义。本研究为SCLC的耐药机制的研究提供了一定的理论基础和未来的研究方向。但MDR的发生机制非常复杂，多种潜在的上下游转录因子均可对SPHK1发挥调控作用，其中有癌基因，也有抑癌基因。因此SPHK1在SCLC耐药中的具体作用机制，尚需进一步研究加以阐明。
